# T-complex measures in bilingual Spanish-English and Turkish-German children and monolingual peers

**DOI:** 10.1371/journal.pone.0171992

**Published:** 2017-03-07

**Authors:** Tanja Rinker, Valerie L. Shafer, Markus Kiefer, Nancy Vidal, Yan H. Yu

**Affiliations:** 1 Department of Linguistics/Zukunftskolleg, University of Konstanz, Konstanz, Germany; 2 The Graduate Center, City University of New York, New York, New York, United States of America; 3 Ulm University, Ulm, Germany; 4 St. Johns University, New York, New York, United States of America; University of Valencia, SPAIN

## Abstract

**Background:**

Lateral temporal neural measures (Na and T-complex Ta and Tb) of the auditory evoked potential (AEP) index maturation of auditory/speech processing. These measures are also sensitive to language experience in adults. This paper examined neural responses to a vowel sound at temporal electrodes in four- to five-year-old Spanish-English bilinguals and English monolinguals and in five- to six-year-old Turkish-German bilinguals and German monolinguals. The goal was to determine whether obligatory AEPs at temporal electrode sites were modulated by language experience. Language experience was defined in terms of monolingual versus bilingual status as well as the amount and quality of the bilingual language experience.

**Method:**

AEPs were recorded at left and right temporal electrode sites to a 250-ms vowel [Ɛ] from 20 monolingual (American)-English and 18 Spanish-English children from New York City, and from 11 Turkish-German and 13 monolingual German children from Ulm, Germany. Language background information and standardized verbal and non-verbal test scores were obtained for the children.

**Results:**

The results revealed differences in temporal AEPs (Na and Ta of the T-complex) between monolingual and bilingual children. Specifically, bilingual children showed smaller and/or later peak amplitudes than the monolingual groups. Ta-amplitude distinguished monolingual and bilingual children best at right electrode sites for both the German and American groups. Amount of experience and type of experience with the target language (English and German) influenced processing.

**Conclusions:**

The finding of reduced amplitudes at the Ta latency for bilingual compared to monolingual children indicates that language specific experience, and not simply maturational factors, influences development of the neural processes underlying the Ta AEP, and suggests that lateral temporal cortex has an important role in language-specific speech perception development.

## Introduction

Bilingualism is defined as the knowledge and use of two languages by an individual. It is characterized by a wide range of abilities in the two languages, from minimal proficiency in one of the languages, to high proficiency in both languages [1]. Many studies have focused on identifying the factors that contribute to the proficiency level achieved in the two languages. These studies show that factors such as age of acquisition (AoA), and amount of experience/use (AoE) contribute to proficiency levels [2,3]. One finding from these studies is that early learners (in childhood) of a second language (L2) typically show better phonological skills in the later-learned language than later learners (after approximately 14 years of age) [4,5]. The poor phonological skills of late learners manifest as more accented production of the L2 and poorer speech perception of L2 phoneme categories that are not in the first language (L1).

Poorer L2 phonology in late L2 learners could be due to a critical or sensitive period for learning the phonology of the language [6]. Learning an L2 later than puberty generally has a striking effect on L2 production and perception. The effect of amount of experience with a language is considered to be markedly weaker than the effect of AoA in the area of phonology. Thus, bilingual adults who have learned a language early in life (particularly, before five years of age), and who actively continue to use both languages are expected to show native-like performance in both languages.

Several recent studies, however, that have used neural measures suggest differences in phonological processing of, at least, one of a bilingual’s two languages, even in early L2 learners. For example, Sebastián-Gallés et al. [7] found that Spanish-Catalan bilinguals, who had learned Spanish first and Catalan around three years of age, did not recognize (via the error related negativity ERP effect) mispronunciations of Catalan words while Catalan-dominant bilinguals did. In another study, Spanish-English bilinguals showed reduced sensitivity to an English vowel contrast compared to monolingual English listeners [8]. This pattern was observed irrespective of whether English had been learned early (before five years of age) or late (after 16 years of age), and despite the early learners showing native-like behavioral speech perception. It is unclear why these early bilinguals showed less robust neural responses to a vowel contrast in the second language (English). Differences in experience with the two languages during these early years could account for some early learners developing less robust processing. Thus, to adequately interpret the impact of early bilingual speech development, it is necessary to examine speech processing during these early years, when the amount and nature of bilingual input can be more easily and accurately determined.

Monolingual children begin learning to perceive phonemes of the ambient language from birth, and effects of this learning can be observed as early as six months of age [9]. Kuhl argues that children begin showing “neural commitment” to the sound patterns of the native language during this early time frame [10]. Given these findings of early learning, it is of considerable interest to determine how children exposed to a second language during childhood acquire a novel set of phonemes, some of which will not match with the L1. As noted above, evidence suggests similarities in phonological processing in a given language for bilingual and monolingual children [11], but some studies have also observed differences [12–14]. A variety of factors, such as the input situation, language similarity, and the age of first exposure, may account for different findings across studies.

Bilingual children who acquire a second language after the first (i.e. after 2 or 3 years of age), and who show no delays in the L1, have successfully acquired one language before learning the second. In the L2, however, they may show delays compared to monolingual learners because they are receiving less exposure to the L2. Unlike clinical populations who show delays in L1 acquisition, these L2 learners are clearly competent in acquiring language. There is no reason to suspect that L2 child learners are poor phonological processors; however, their L2 speech perception may still be delayed or different because they have less experience with the L2, and this experience may be of a different nature.

Investigations of speech perception in these early child L2 learners (before five years of age) have resulted in inconsistent results. A few studies of L2 learning during preschool suggest that as little as two months of input can lead to native-like neural discrimination, at least for some phonological contrasts [15,16]. The findings of Rinker et al. [17], however, indicate that two years of input may be insufficient to develop robust phonological categories. They suggested that the L2 phonological input (in this case, German) to the bilingual children (with Turkish as an L1) may have been of insufficient quantity (e.g. in the daycare setting, but not at home), and thus led to poorer neural discrimination of vowel categories in the L2.

Studies examining phonological abilities of bilingual children using working memory tasks, such as non-word repetition, also indicate that performance is related to the amount of experience with the languages [18]. It is possible that the quality (specifically, accentedness of the L2 input) as well as quantity of the input in the bilingual environment could underlie apparent poorer performance on phonological tasks in an L2.

Neural measures of speech processing appear to be particularly sensitive to differences in L2 phonological processing as they sometimes reveal differences in processing that are not observed at the behavioral level [7,8]. Previous neurophysiological studies have primarily used the mismatch negativity (MMN) event-related potential measure to examine L2 speech discrimination. MMN reflects acoustic change detection, but is also modulated by phonological experience. MMN has good sensitivity for detecting language impairment, but poorer specificity [19]. For these reasons it is useful to examine alternative neural measures that index other types of processing, such as auditory encoding and that have the potential to be obtained in shorter testing sessions.

Recent studies have demonstrated that neural measures of auditory encoding recorded from temporal electrode sites may be sensitive to language abilities at the level of encoding auditory information. These temporal site measures have been called “the T-complex”. Several studies found poorer T-complex measures in children with language impairments than in age-matched peers with good language skills [19, 20]. However, it is yet unclear if these temporal-site measures index general speech processing abilities or language-specific experience with speech sounds. If the T-complex reflects language-specific experience, then differences would be observed for L2 learners of a language who have not yet fully acquired the L2 phonological categories. If the T-complex reflects more general abilities then no difference would be observed for monolingual and bilingual children.

### T-complex

The T-complex measures are auditory evoked potentials (AEPs) recorded at lateral temporal electrode sites and were first examined by Wolpaw and Penry [21] in healthy adults. AEP responses from the temporal sites T3 and T4 (T7 and T8, respectively in the newer 10–10 notation) were elicited to a series of clicks in a time window between 75 and 225 ms. The peaks were labeled Ta, a positive peak between 105 and 110 ms, and Tb, a negative peak between 150 and 160 ms. An earlier Na peak, which was not measured in Wolpaw and Penry [21] can also be observed at temporal sites. In children, Na may partially reflect the opposite pole of the positive peak (P1) at superior sites [22]. The Ta and Tb peaks were clearly distinct from N1 and P2 responses recorded at the vertex (Cz) [21]. The authors suggested that the N1 and P2 activity was the result of sources in auditory cortex from both hemispheres, in part, because it was more wide-spread across the superior surface of the scalp. They also hypothesized that the activity underlying the T-complex might be generated in secondary auditory cortex in each hemisphere. Dipole modeling in a later study supported their claim that the neural generators of the T-complex can be traced to bilateral radial dipoles in the temporal lobe. This source orientation is consistent with secondary auditory cortex activity [23].

The T-complex shows an asymmetrical pattern over the left and right sites. Several studies have found that the Ta is more prominent over the right than the left hemisphere [14,20,22]. Only one study suggests that Na and Tb are more prominent on the left side [24]. A study that directly compared speech (vowel) and non-speech (1000-Hz tone) processing found greater activation over the left temporal site at latencies following the N1 (and magnetic N1) for the speech compared to non-speech but no differences over the right temporal site [25]. This finding suggests a special role for auditory cortex underlying the left temporal site in processing speech.

Several studies have shown that T-complex peak amplitudes can serve as an index of robustness of auditory/speech processing [14, 20, 26, 27]. The Ta peak appears to be the most sensitive to language deficits. Ta amplitude was attenuated in children with Specific Language Impairment SLI ([14],[20]). In addition, a study by Groen et al. [26] found atypical lateralization of Ta amplitudes in children with Down’s Syndrome. Both Bishop et al. [20] and Shafer et al. [14] found that children with SLI exhibited attenuated neural responses to speech (and tones [20]) and to a wide variety of linguistic stimuli (vowels, real words, nonsense words, function word “the” [14]). The general morphology of the waveform at temporal sites in the latency range from Na to Tb also has been shown to differ for children with SLI compared to controls with typical language [14, 28]. A few studies have also observed delays in the Tb latency in children with autism [29, 30]. Thus, Ta and Tb peaks may serve as indices of speech and language development.

Maturational studies of the T-complex peaks suggest that the attenuated Ta peak amplitude in children with disorders could be the result of delayed maturation. A recent study by Shafer et al. [22] revealed that only Na is consistently present to a vowel stimulus in children under four years of age. The Ta peak emerges between four and eight years of age. Tb was not easily identified in the children’s data (the oldest child had just turned eight years of age), but was found in adults. Studies of T-complex maturation across grade-school and into adulthood suggest that the Ta peak amplitude first increases from approximately seven to 11 years of age, and then decreases in amplitude up to adulthood [24, 31, 32]. These findings, taken together, suggest that the generators underlying the Ta and Tb peak are highly immature before four years of age. Additionally, the generators may have a different orientation in infants and toddlers, leading to absence of the peaks at the temporal sites T7 and T8 [22]. There was some evidence that the Na peak reflected the opposite pole of the fronto-central P1, which dominates young children’s ERP waveforms in studies where the auditory stimuli are presented at relatively fast rates (faster than one per second) [22].

What is not known is whether the emergence of the T-complex around four years of age is also influenced by language experience. To date, one study has analyzed the T-complex in relation to language experience in adults [33]. This study compared responses from Polish-English bilinguals and English monolinguals elicited to a Polish-specific phonotactic feature of the cluster “pt” (/pt/ can occur at the onset and offset of Polish words, but only at the offset of English words). The P1-N1-P2 complex measured at frontal sites was not affected by linguistic experience. However, temporal sites showed increased negativity of the ERP from 40 to 246 ms for the “pt” compared to “p-schwa-t” onsets only in Polish adults. The English participants showed no difference between phonetic onsets. The increased negativity was a general effect across this time span, encompassing all three temporal site peaks (Na, Ta and Tb). The amplitudes of the specific peaks were not analyzed in this study because the signal/noise was not sufficient (too few trials) to identify peaks in individual participants.

### The present study

In the current report, we examined whether the neural responses at temporal sites (namely, the T-complex) were sensitive to language experience in children with typical development that grew up with either one or two languages. Two different experiments were performed, which were designed independently of each other, but were similar in focusing on L2 vowel processing in preschool children. Both studies examined processing of highly similar vowels as target stimuli. These similarities justified examining the data in the same report rather than in two independent papers, as will be further explained below.

Monolingual and bilingual children from two different language cultures (United States of America, USA, and Germany) participated. Bilingual Spanish-English children from the New York City area, USA, often live in communities that are dominated by Latino culture. These children may receive mostly Spanish in the home, but once the child enters the schools, English is the dominant language of education. Some of the children participate in bilingual Spanish-English classrooms, which provide half of the education experience in Spanish and half in English. With increasing age, the amount of English use in the school increases relative to Spanish, even in bilingual programs. Thus, many of these bilingual children will shift from dominant experience in Spanish before approximately four years of age to mostly English in school after this age. The children come from a range of Latino backgrounds, with the largest groups from Dominican Republic, Mexico, and South America (Colombia, Ecuador, and Venezuela).

Turkish-German children served as the bilingual population from Germany. They were from a relatively homogeneous background in Ulm, which is a medium-sized city (120,000 inhabitants) in southern Germany. Both parents were of Turkish origin. Turkish is typically the dominant language in the home and environment of Turkish children raised in Germany until the child attends daycare. In this daycare setting, German is typically the only language in use.

By using two groups from two different language backgrounds, all in the year before school entry (which is one year later in Germany, hence an older group was examined), it was possible to explore similarities and differences across subject groups. In both studies, neural responses to the vowel phoneme /Ɛ/, which is found in both German and English, were examined. The phoneme /Ɛ/ is perceived as a variant (allophone) of the Turkish /e/ for L1 Turkish listeners and as a variant of Spanish /e/ for L1 Spanish listeners. More specifically, Turkish and Spanish listeners have difficulty categorizing /e/ and /Ɛ/ as two different vowels because the [Ɛ] phone does not lead to a word meaning change. The [Ɛ] phone has a higher first formant and lower second formant than /e/, but is acoustically closer to /e/ than /a/. (/a/ has a higher F1 and lower F2 than [Ɛ]). Early experience with English (before five years of age) for Spanish-English adult bilinguals can allow for good categorization of these English phonemes [8].

We hypothesized that experience with a language having /Ɛ/ as a distinct phoneme from /e/ would have an effect on the T-complex measures at the temporal sites. Specifically, we expected that the L2 learners of English and German would show differences in the T-complex measures because they have had less experience with the language-specific phonetic realization of the /Ɛ/ phoneme. This effect could be seen as a reduction and/or delay in latency of the T-complex peaks (Ta and Tb) compared to monolingual children. We particularly expected that the Ta peak would show less positive amplitude for bilinguals than monolinguals, based on findings from children with SLI, but only if the pattern observed in children with SLI was the result of poorly formed or inaccurate phonetic detail for phonological categories. We also predicted that experience with the L2 would show a positive relationship with the Ta peak measure. Thus, we expected child L2 learners to show T-complex measures increasingly like their L1 peers, as they gained more L2 experience. This hypothesis was tested by examining the relationship between T-complex measures and language input measures obtained via questionnaires regarding language input and experience, which were filled out by parents/caretakers of the children.

## Methods

### US participants

Eighteen Spanish-English children (7 females) and 20 monolingual American-English children (7 female) were examined. The mean age of the monolingual children was 4;8 years of age. Bilingual children were on average 4;7 years of age. Children were recruited via letters sent to the home. The letter explained the nature of the study and invited the parent/guardian to contact the laboratory to learn more about the study using email, telephone, or by mailing back a postcard including contact information. The study was then explained to the caretaker over the phone and a laboratory visit was scheduled. In the laboratory, the experimental measures were explained to the parent and child. The parent/guardian signed a consent form and children gave verbal assent. The study was conducted in accordance with the Declaration of Helsinki and was approved by the City University of New York, Internal Review Board (IRB) ethics committee.

The two groups did not differ with respect to language measures on the Preschool Language Scale-4 (PLS) in English for Expressive or Receptive Language [34]. The American-English group tended to have larger vocabularies, as measured on the English Peabody Picture Vocabulary-3 (PPVT) (see Table 1) [35]. The bilingual children were also tested on the Spanish version of the PPVT, the Spanish Test de Vocabulario en Imagenes Peabody (TVIP) [36]. The PPVT and TVIP are standardized to have a mean of 100 and SD of 15. This measure was chosen to test language abilities because standardized measures were available for both languages. Vocabulary knowledge also provides an indirect measure of language experience, since the test is designed to assess knowledge that a child is likely to have in age-appropriate situations. The items on the test are delivered in the order of higher frequency/familiar to lower frequency/less familiar. Thus, a low score on either test suggests insufficient input in that language. In addition, low scores on both tests indicate risk for language impairment. Bilingual children’s scores ranged from high word knowledge (score of 142) to those who knew only a few words in Spanish (scores of 50). All children were screened for hearing and language (see Table 1). No child had poor language scores on both English and Spanish language tests (i.e., scores more than 1 SD below the standardized mean).

**Table 1:** Subject characteristics. US-Study: PLS = Preschool Language Scale-4 (mean: 100), PPVT = English Peabody Picture Vocabulary-3, TVIP: Test de Vocabulario en Imagenes Peabody. *Six of the bilingual group only have English PPVT scores. German study: receptive, expressive and pseudo-word repetition skills are displayed with T-scores (a T-score between 40–60 is considered within the normal range). Turkish lexical test: Max. score: 60.

	English	Spanish-English	Difference		German	Turkish-German	Difference
Number	20	18			13	11	
Age in months	58 (7.6)	57 (6.6)	p = .82		65 (4.2)	62.2 (5.6)	p = .17
PLS receptive	112 (13)	106 (18)	p = .16	**Receptive language**	51.5 (7.8)	45.4 (10.1)	p = .10
PLS expressive	110 (11)	104 (18)	p = .22	**Expressive language**	54.9 (7.4)	39.5 (7.7)	p = .00
PPVT	113 (14)	105 (17)	p = .08	**Pseudo-word repet.**	44.4 (5.8)	42.8 (8.1)	p = .58
TVIP		77 (32) range: 142 to 50.		**Turkish lexical test**		39.2 (6.1)	

#### Language background questionnaire

The parent/guardian of bilingual children was asked to complete a language background questionnaire (LBQ, available from the authors on request). This questionnaire revealed that eight of the Spanish-English children showed balanced exposure to/use of Spanish and English, nine were dominant in English, and only one was dominant in the use of Spanish at the time of the study. From the mothers, 28% of the bilingual children heard only Spanish, 33% heard both Spanish and English and 39% heard only English. From the fathers, 17% heard only Spanish, 39% heard both Spanish and English and 44% heard only English. Only one child exclusively heard Spanish from birth from both the mother and the father but this child had older siblings who spoke English. Thus, all children heard some English from birth. Four of the bilingual children had English-speaking parents and were exposed to Spanish via Nanny, sitter or daycare. Nine of the children in the bilingual group received mostly English (LBQ means greater than 2, on a scale from -3 (all Spanish) to 3 (all English)) and demonstrated little knowledge of Spanish vocabulary on the standardized test of vocabulary. However, it is important to recognize that the TVIP is designed to test children with monolingual input in Spanish, and a poor score does not necessarily mean that a child has no knowledge of Spanish.

### German participants

Thirteen German (mean age: 5;4 years, 5 female) and 11 Turkish-German children (mean age 5;1 years, 5 female) were recruited from local day care centers in the city of Ulm, Germany. Written consent was obtained from the parents. The study was conducted in accordance with the Declaration of Helsinki and was approved by the Ethics Committee of the Ulm University.

All children had non-verbal standard IQ scores above 85 [37] and normal hearing. Groups did not differ significantly regarding age or IQ, receptive language skills or pseudoword repetition. The groups differed, however, significantly in their productive German language skills as measured by the productive subtest of a German language development test [38]. In this standardized language development test, children have to repeat sentences with increasing complexity (expressive skills) or re-enact a sentence using wooden toys (receptive skills). In a passive lexical test of Turkish language skills (part one of CITO, Arnheim, NL) Turkish—German children scored 39 out of 60 points (“satisfactory”) (see Table 1). This test is computer-based and children have to click on the correct picture out of four.

#### Language background questionnaire

A language and developmental questionnaire (available from the authors on request) showed that all but one of the Turkish—German children were born in Germany. The Turkish—German child that was not born in Germany had lived in Germany for the previous four years. Their mean age of acquisition of German was 28.8 months (SD 11.6). Fifty-four percent of the mothers reported Turkish-dominance, 27% German-dominance, and 18% reported equal proficiency in both Turkish and German. Of the fathers, 70% reported Turkish-dominance, 30% reported equal proficiency in both languages.

On average, children entered day care (which is equivalent to preschool in the United States) at the age of 37 months (SD 11.3). Eight children attended day care for half a day, and three children attended day care full time. Thus, all children spend at least half a day in a predominantly German-speaking environment. Within the family, the fathers spoke mostly Turkish with the child (66% of the fathers spoke Turkish, 33% both languages). Forty-four percent of the mothers use Turkish, 44% German, and the rest of the mothers use both languages. Of the siblings interacting with the child, 50% use both languages, 25% Turkish only, and 25% German only.

The monolingual German children were not exposed to a second language at home. For all children attending preschool, German was the primary language of teachers and most of the children.

### Stimuli and recording

#### Study 1

**Fig 1:**
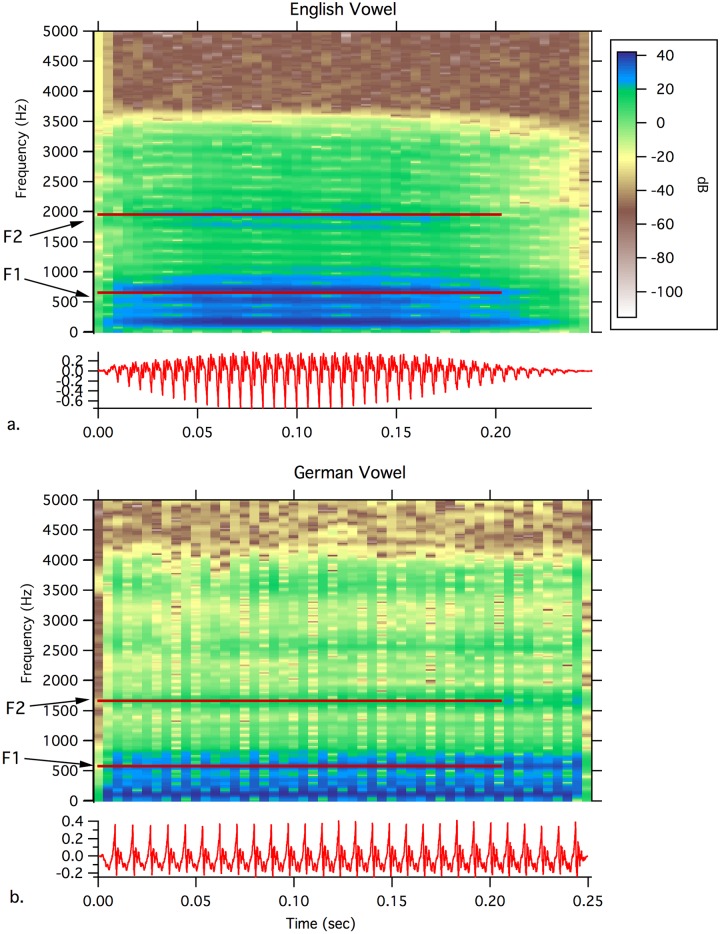
Spectrograms for the English and German vowels. a) The center formant frequencies for English [Ɛ] are shown by the red lines and were F1 = 650 Hz and F2 = 1950 Hz. b) The center formant frequencies for German [Ɛ] were F1 = 570 Hz and F2 = 1660 Hz. F1 and F2 measurements were made using Praat. Figures were created using IGOR 7, Wavemetrics Inc.

**Stimuli and Procedure**: The stimulus was a vowel [Ɛ] resynthesized from a natural vowel. The F1 and F2 formant frequencies were edited to produce a continuum of 9 stimuli from the vowel [Ɛ] in “bet” to the vowel [I] in “bit (Analysis-by-synthesis Lab (ASL), 3.2, [14]). The ASL method uses a similar procedure to that described for creating the German vowels (see below). The vowel duration was edited to 250 ms. As shown in Fig 1a, the amplitude increased over the first 60 ms, and then began to fall from 120 ms to the end of the stimulus, reflecting the amplitude envelop of naturally-produced vowels. The fundamental frequency (F0) was maintained at 179 Hz for the first 60 ms and then gradually fell to 165 Hz. The F1 center frequency was 650 Hz and F2 center frequency was 1980 Hz. This vowel stimulus was perceived as [Ɛ] by children and served as the standard stimulus [39]. A deviant stimulus [I] was delivered on 20% of the trials, but will not be examined in the current analysis (see [14]) Stimuli were presented at a rate of 1 stimulus per 650 ms (ISI: 400 ms) within a sequence of 10 stimuli (eight standards and two deviants). The sequence of 10 was separated by an ISI of 1500 ms. Stimuli were presented in free field at 86.5 dB SPL. One speaker was placed directly in front of the children (approximately 1 meter at eye level). A second speaker was suspended from the ceiling approximately 1 meter behind and above (45 degree angle). A total of 2000 stimuli (1600 standards) was presented. Children watched a self-selected video with the sound muted. The study took approximately 45 minutes for stimulus delivery and 15–20 minutes for electrode net application.

**Instrumentation and EEG data processing**: The same instrumentation and data processing parameters were used in this study as in a study of the T-complex in 3-month-old to 7-year-old children [22]. Furthermore, the 4- to 5-year-old monolingual child data are part of this prior report. A 65-channel Geodesic net with electrodes attached to sponges was used. Two of the electrodes beneath the eyes were used to monitor eye movements and the vertex served as the reference during data collection. The net was first soaked in a saline solution of potassium chloride and then placed on the child’s head. Impedances were maintained below 40 kΩ, which was acceptable for the high-input impedance Geodesic amplifiers (200 MΩ input impedance). The EEG was amplified and filtered with a bandpass of 0.1 to 30 Hz. The data were acquired using NetStation software (version 4.1.2) at a sampling rate of 250 Hz per channel. The EEG was processed off-line, using a low-pass filter of 20 Hz, and segmented into epochs with an analysis time of 800 ms poststimulus and 100 ms prestimulus. Epochs were baseline corrected (100 ms, pre-stimulus) and then examined for artifact using Netstation. Epochs with excessive artifact (if the differential average amplitude was greater than 100 μV in 5 channels, if the fast average was greater than 200 μV, or if a channel showed zero variance) were excluded from the average. Bad channels (on 20% of the trials) were replaced by a spline interpolation algorithm from surrounding channels. Data were re-referenced to an average reference. Responses to the [Ɛ] stimulus, including only those epochs which occurred in the fourth through 10^th^ position of the sequence, and that did not follow a deviant ([I]) stimulus were averaged. The majority of participants had between 450 and 900 artifact-free EEG segments (except two, who had 268 and 351 segments).

#### Study 2

**Stimuli and Procedures**: The stimulus was the vowel [Ɛ], created by the Semi-synthetic Speech Generation method (SSG [40]), and was based on vocal tract models from sustained /Ɛ/ produced by a German speaker. (The investigation of the MMN-response to the vowel contrast vowel contrast /Ɛ/ versus /e/ was the aim of a previous study [17]). The duration of the stimulus was 250 ms. A 5-ms rise and fall time was applied at the beginning and the end of the stimulus waveform using Hanning-windowing. Amplitude was maintained at the same level (fluctuating by less than 1.2 dB) for the remainder of the stimulus. The Fundamental frequency was 115 Hz and F1 and F2 were 570 Hz and 1660 Hz, respectively (see Fig 1b). The sounds were presented through headphones at 90 dB SPL. The ISI was 650 ms. A total of 595 stimuli was presented.

Children watched a cartoon movie and were told to focus on the movie. As this experiment was part of a larger study including four experimental conditions (about 10 min. each), the total testing time was about one hour including breaks.

**Recording and processing of the data**: The same processing parameters were used as for the published report examining vowel discrimination in Turkish-German children [17]. The EEG was recorded from 39 electrodes using the BrainAmp amplifiers (Brain Products, Gilching, Germany). Eye movements were monitored with electro-oculogram (EOG) electrodes attached below and at the outer canthus of the left eye. The reference was to the left earlobe. Electrode impedance was kept below 10 kΩ. The EEG was sampled at a rate of 500 Hz (band pass filter was at 0.1–70 Hz). The offline analysis was conducted using Brain Vision Analyzer (2.0) (Brain Products, Gilching, Germany). First, EEG-data was high-pass (0.1 Hz) and low-pass filtered (16 Hz). A linear derivation served to combine eye electrodes into VEOG and HEOG. Artifacts induced by eye movements were corrected using independent- component-analysis [41]. Data were then segmented (-100 to 700 ms) and baseline-corrected (pre-stimulus baseline of 100 ms). Residual artifacts in the EEG-data (activity exceeding 70 μV) using artifact rejection were excluded from further analysis. Artifact-free EEG segments were averaged for the /Ɛ/ stimulus. Averaged data were re-referenced from the nose to an average reference for comparability with the American Study. For the purpose of this study, only the standard stimulus was analyzed while the deviant /e/ was not.

## Analysis

**Fig 2:**
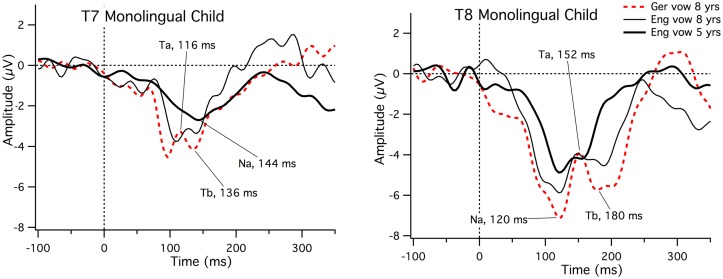
ERPs at T7 and T8 for the German vowel and English vowel using the American instrumentation and free field stimulus presentation with an 8-year old monolingual child. The same child had participated as a 5-year old in the American study.

First, three time windows were selected from the grand average data at electrodes T7 and T8 to constrain selection of individual peaks. As there are not yet any established time windows for children, the following procedure was undertaken: The individual peak choices were based on visual inspection and these had to be confirmed by the first and second authors. For the American study, Na was defined as a negative peak occurring in the range of 60–150 ms, Ta as a positive peak in the range of 100–200 ms, and Tb as a negative peak between 150–250 ms. For the German study 30 ms were subtracted from these values as responses occurred 30 ms earlier. There were several possible reasons for the latency difference between studies, including stimulus differences, instrumentation differences, stimulus delivery differences (headphone versus free-field) and age differences. Other studies suggest that free field versus headphone presentation may account for some of the difference (compare values from this study with earlier values in [14, 20, 26], see [22] for a discussion of the effect of ear inserts versus headphones). However, rather than merely speculating regarding the cause of the latency differences, we tested one 8-year old child on both the German and US vowel stimuli using the US instrumentation and free field presentation of the vowels to allow us to evaluate why there was a 30 ms latency difference. This child had participated in the American study as a 5 year old and was available for retesting. Fig 2 illustrates that the latencies to the German and the English vowel are highly similar at the right site (T8 Na = 120 ms for both vowels, T8 Ta = 152 ms to both vowels and T8 Tb = 136 ms for German and 140 ms for the English vowel) and approximately 10 ms later for the English compared to German vowel at the left site (T7 German Na = 96 ms, Ta = 116 ms and Tb = 136 ms; English Na = 108 ms, Ta = 128 ms and Tb = 140 ms). The figure also illustrates larger amplitude peaks to the German than the English vowel. This is likely to be related to the more abrupt rise time for the German compared to English vowel. It is also clear from this figure that Ta and Tb can be clearly identified to the English vowel at 8 years of age but not at five years of age. Overall, T-complex morphology was not affected by the different laboratory set-ups.

Na and Tb were chosen as the most negative-going peaks by visual inspection in the defined time range and Ta as the most positive-going peak (thus, the amplitude value of a positive-going peak could be negative and vice versa) by the first two authors. If two peaks were identical in amplitude, the earlier one was chosen. Peaks needed to be in sequence: Na, Ta, Tb. If there was no clear peak, it was searched for outside the window, but marked as late or early (and not included in the statistical analysis as indicated below). The proportion of identifiable peaks was calculated. Latencies and amplitudes were then measured for the three peaks for all the clear cases. Fig 2 (above) illustrates that in some cases no clear peak was observed, particularly for Ta and Tb (see left site trace for the 5 year old in Fig 2).

Mixed models logistic regression analyses were used to evaluate whether the presence versus absence of a T-complex peak or the amplitude of a T-complex peak would predict the monolingual/bilingual status of a participant. Logistic regression is ideal for testing categorical variables and has the advantage of no assumptions regarding the linearity, normality, homoscedasticity, and measurement level of the independent variable ([42]. We used R ([43]) and lme4 ([44]) to perform mixed-effects binary logistic regression analyses. The Akaike Information Criterion (AIC) and Liklihood Ratio were used to compare the fit of competing models.

To determine whether the morphology of the T-complex waveforms differed between groups, Pearson’s product moment correlations were calculated between each individual child’s waveform and the monolingual grand mean (compare to [14]) and then the correlation coefficients were compared between groups. For the American data, 55 points between 80 and 300 ms were used in the correlations. For the German data, 110 points between 50 and 270 ms were used (recall that these data were collected at a higher sampling rate).

### Relationship with behavioral measures

Pearson’s product moment correlation was used to examine the relationship between temporal site ERP measures and behavioral and language background results. These comparisons included the expressive and receptive language tests as well as some of the language background variables (including amount of exposure to the different languages, parent’s language dominance and self-rated skills, and amount of use by parent, siblings and caretakers).

## Results

**Fig 3:**
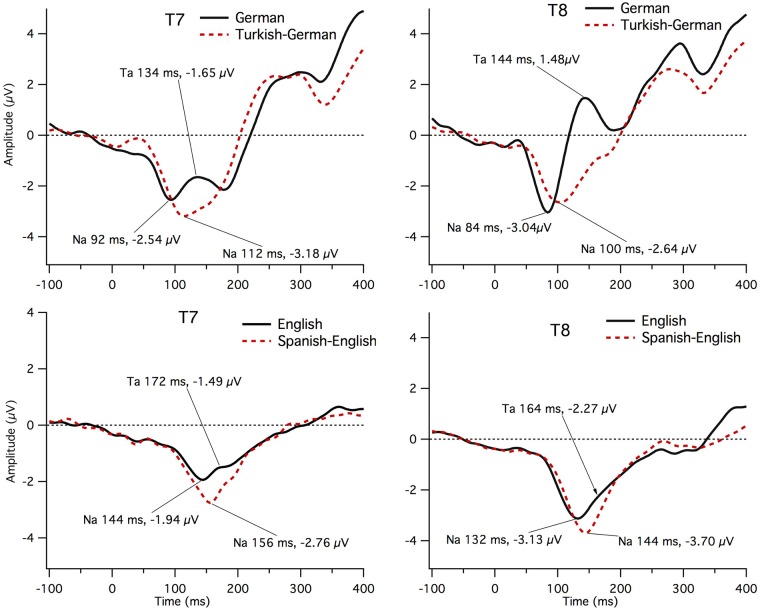
Grand mean ERPs in each group from left (T7) and right (T8) sites in monolingual and bilingual children. The top graphs show children from the German sample and the bottom graphs from the American sample. Na, Ta and Tb peaks are labeled. Data are shown with an average reference.

Graphs of the grand mean ERPs comparing the monolingual and bilingual children at T7 and T8 are displayed in Fig 3. Na was the most easily identified peak for all subjects as can be seen by the prominent negative peak for all four groups at both left (T7) and right (T8) sites in Fig 3. The grand mean Na is later for both bilingual groups compared to their monolingual counterparts. For the American-English (AE) monolingual Grand Means, Ta was better defined at the left site, T7, than the right, T8, site. Tb was not clearly visible for the monolingual AE group, but a small deflection following Ta was apparent at the left site. For the bilingual Spanish-English grand means, neither Ta nor Tb were clearly defined. For the German monolingual group Ta was more prominent on the right than the left site but clearly visible at both sites. Tb was clearly elicited in the German monolingual group at both left and right sites. The Turkish-German bilinguals did not show prominent Ta or Tb peaks in the grand means.

### Identifiable peaks

Peaks were defined as “identifiable” if there was a visible negative or positive peak within these pre-defined time windows (see Analysis). There were differences across groups in the number of identifiable peaks for individuals (compare Table 2). The right (T8) Na peak was the most reliably present peak across groups (over 94% for both sites and groups). In contrast, the left Ta in the Turkish-German group (45% identifiable) and right Ta in the Spanish-English group (39% identifiable) were not clearly-defined for over half of the children in these groups. For Tb, the right site peak was not identifiable for many children in both the English group (35%) and the Spanish-English group (56%). The Tb peak was absent for many German-Turkish bilinguals at the left site (36%).

**Table 2:** Percentage of identifiable peaks for each group at the right (T8) and left (T7) sites for Na, Ta and Tb.

	Right Na	Ta	Tb	Left Na	Ta	Tb
Mono English	95	75	65	80	75	65
Spanish-English	94	39	44	56	65	61
Mono German	100	100	85	85	92	92
Turkish-German	100	82	82	91	45	64

A mixed-effects binary logistic regression analysis was performed to examine the relationship between the bilingual/monolingual status and the presence/absence of a Na-Ta-Tb peak. The predicted variable was bilingual/monolingual status. Spanish-English and Turkish-German children were collapsed into a single, bilingual group and English and German monolingual children into the monolingual group. The fixed effects include presence/absence of peak, peak components (Na, Ta and Tb), and hemisphere (left and right). Participant was the random effect. The Stepwise method was used, and the fitness of the null model and all the other models in a likelihood ratio test was compared. Sequential tests were conducted to compare the change in deviance between two fitted models, and the *p*-value is computed by comparing the amount of change in deviance using chi-square distribution with the degree of freedom equal to the change in degree of freedom for the two comparison models [45]. Adding peak presence status significantly improved the model, but adding hemisphere and time as fixed effects or random effects to the fitting models did not reduced the residual. The best model consisted of participant as the random effect, and presence/absence of peak as the fixed effect. Monolingual participants were more likely to have Na-Ta-Tb peaks (χ2 (1) = 4.79, p = 0.03, AIC = 515.4). Table 3 shows the results from the mixed effects logistic regression with all the independent variables. Although hemisphere and the Ta and Tb subcomponents were nonsignificant predictors, we included them in the table because these two factors are related to the purpose of our study. In the final model, presence of peak decreased the odds of being a bilingual by 47%.

**Table 3:** Results of the mixed effects logistic regression analysis on the presence versus absence of Na, Ta and Tb peaks.

Predictor	Coefficient	SE	Z	Odds ratio	95% CI	*p*
Intercept	0.47	0.34	1.39	1.59	[0.83, 3.09]	0.16
Peak	-0.63	0.28	-2.27	0.53	[0.31, 0.91]	0.02*
Hemisphere(Right)	0.02	0.21	0.10	1.02	[0.68, 1.54]	0.92
Peak = Ta	-0.12	0.26	-0.47	0.88	[0.53, 1.48]	0.64
Peak = Tb	-0.17	0.27	-0.63	0.84	[0.50, 1.42]	0.53
	Null deviance: 514.15 on 371 degrees of freedom					
	Residual deviance: 508.93 on 367 degrees of freedom					
	AIC: 519					

*denotes a significant result at level p < 0.05

### Amplitude measures

Peak amplitude values could not be obtained for a large proportion of the participants, and, thus, the analysis of amplitude focused on amplitude across successive time intervals. All participants were included in these analyses. Table 4 (American groups) and Table 5 (German groups) present the mean amplitude for 20-ms intervals spanning the Na, Ta and Tb peaks.

**Table 4:** Mean amplitudes in μV (SD in parentheses) for the American groups at left and right electrode sites.

	80–100 ms	100–120 ms	120–140 ms	140–160 ms	160-180ms	180–200 ms	200–220 ms
Left Monolingual English	.68 (.59)	-.89 (.89)	1.43 (1.54)	-1.88 (2.15)	-1.69 (2.09)	-1.46 (1.79)	-1.24 (1.33)
Left Spanish-English	-.74 (1.49)	-1.02 (1.74)	-1.69 (2.05)	-2.38 (2.22)	-2.65 (2.17)	-2.12 (1.62)	-1.56 (1.35)
Right Monolingual English	-.88 (1.01)	-1.89 (1.38)	-2.89 (2.12)	-2.99 (2.38)	-2.39 (2.33)	-1.86 (2.01)	-1.41 (1.58
Right Spanish-English	-.78 (1.14)	-1.52 (1.35)	-2.76 (1.59)	-3.61 (2.24)	-3.22 (2.31)	-2.20 (2.05)	-1.37 (1.48)

**Table 5:** Mean amplitudes in μV (SD in parentheses) for the German group at left and right electrode sites.

	60-80ms	80–100 ms	100–120 ms	120–140 ms	140–160 ms	160–180 ms	180–200 ms
Left Monolingual German	- 1.05 (1.24)	-2.09(1.90)	-2.42(1.77)	-1.87(1.53)	-1.68(1.29)	-1.89(1.37)	-2.08(1.89)
Left Turkish-German	-.54 (.99)	-1.62(1.35)	-2.82(1.95)	-3.14 (1.80)	-2.89(1.33)	2.52(1.13)	-1.69(1.41)
Right Monolingual German	-1.55 (1.06)	-2.85(1.16)	-1.97(2.19)	.18(3.48)	1.37(3.44)	1.06(2.85)	.34(2.54)
Right Turkish-German	-.87 (.80)	-2.03(1.16)	-2.60(1.33)	-2.36(1.08)	-1.64(1.37)	-1.01(1.30)	-.71(1.33)

A mixed-effects binary logistic regression was used to examine the relationship between the bilingual/monolingual status and amplitude. This analysis addressed whether the amplitude of the response predicted bilingual/monolingual status. Spanish-English and Turkish-German children were collapsed into a single, bilingual group and English and German monolingual children into the monolingual group. We started with participant as the random effect, and fit the models stepwise by entering the amplitude as the fixed effect and participant as the random effect. Time window (80 to 220 ms for the American English group and 20 ms earlier for the German groups) and hemisphere (left and right) were also entered stepwise as fixed effects, and as nested factors under the random effect participants. We compared the fitness of the models in a likelihood ratio test. Adding amplitude to the null model significantly reduced the residual, but adding hemisphere and time as fixed effects or random effects to the fitting models did not reduce the residual. Adding the interactions (amplitude x hemisphere, amplitude x time, or amplitude x hemisphere x time) did not reduce the residual. The best model consisted of participant as the random effect and amplitude as the fixed effect, with amplitude predicting language status (χ2 (1) = 11.03, p < 0.001, AIC = 1192.7). This finding indicated that for an amplitude increase of 1 μV, the odds of being a bilingual decreased by 12%. This pattern is evident in Fig 2, above, which shows more positivity for the monolingual than bilingual group across the Na-Ta-Tb time range. Table 6 presents results from the mixed effects logistic regression analysis with all the independent variables in the model. We left two nonsignificant predictors (time and hemisphere) in the final model because these two factors were related to the hypothesis of the study.

**Table 6:** Mixed effects logistic regression analysis on ERP amplitude at the left (T7) and right (T8) sites. Amplitude, hemisphere and time are used as fixed factors. „Amplitude”reflects the amplitude value for the bilinguals for 80–100 ms in the American groups and 60–80 ms in the German groups. Each subsequent time interval is 20 ms later.

Predictor	Coefficient	SE	Z	Odds ratio	95% CI	*P*
Intercept	-0.22	0.19	-1.12	0.80	[0.55, 1.18]	0.26
Amplitude	-0.12	0.04	-3.35	0.88	[.82, .95]	<0.001*
Hemisphere (Right)	<0.001	0.14	0.002	1.00	[.76, 1.31]	1
Time 2	-0.07	0.26	-0.28	0.93	[.56, 1.54]	0.78
Time 3	-0.13	0.26	-0.50	0.88	[.53, 1.46]	0.62
Time 4	-0.16	0.26	-0.61	0.85	[.51, 1.42]	0.54
Time 5	-0.17	0.26	-0.66	0.84	[.50, 1.41]	0.51
Time 6	-0.15	0.26	-0.57	0.86	[.52, 1.44]	0.57
Time 7	-0.10	0.26	-0.39	0.90	[.54, 1.50]	0.69
	Null deviance: 1199.7 on 867 degrees of freedom					
	Residual deviance: 1188.0 on 859 degrees of freedom					
	AIC: 1206					

*denotes a significant result at level p.< 0.05

In summary, a consistent pattern was found for comparisons between monolingual and bilingual groups. Specifically, more children from the bilingual groups were missing peaks. The amplitude of the response was more negative for bilinguals compared to monolinguals in both groups and the pattern did not differ across hemispheres.

### Waveform morphology correlations

The morphology of waveforms of the individuals was compared to the respective grand means of the monolingual groups. Overall, there were no differences between monolingual and bilingual groups in the morphology of the T-complex waveform. However, yet again, correlations were higher for the German monolingual group compared to the English monolingual groups. At T7, 13 out of 20 monolingual English children showed a correlation of *r* > 0.59 with the monolingual grand mean (mean = 0.43, SD = 0.59). Only four showed correlations above 0.87. Seventeen out of the 19 Spanish-English participants showed correlations above 0.59 (mean 0.70, SD = 0.39) with seven showing a correlation greater than 0.87. The reason for the low mean correlation for the monolingual children is that four of the children showed high negative correlations (between -0.42 and -0.69), but only one Spanish child showed a high negative correlation (*r* = -0.75). At T8, 13 out of 20 monolingual English showed a correlation of *r* > 0.57 (mean = 0.52 SD = 0.58) and nine showed correlations over 0.87. Twelve out of 19 bilinguals (mean = 0.51 SD = 0.58) showed correlations greater than 0.57 and nine over 0.87. Three monolinguals and two bilinguals showed high negativity correlations (-0.52 to -0.82). A chi-square analysis showed no significant difference between groups for any of comparisons (*p* > 0.05).

For T7, most children from both groups with low correlations to the Grand Means showed some missing identifiable peaks. For T8, the picture was different. All 7 monolinguals with poor correlations to the Grand Mean exhibited clearly definable peaks. This was somewhat true for the bilingual children as well. Thus, the reason for low correlations in some children was related to latency of the peaks, rather than to presence/absence.

Only two out of the 13 monolingual Germans and none of the Turkish-German bilinguals showed poor correlations at T7. At T8, two Germans and two bilingual Turkish-Germans showed correlations below *r* = 0.5. Chi-square analysis revealed no significant differences between groups.

Interestingly, for the German/Turkish and German groups, the children with low correlations did show at least two clear peaks. So again, poor correlation to the Grand Means for the children was related to latency of peaks rather than to absence of these peaks.

#### Relationship between language experience and behavioral measures across different time windows

For the Spanish-English bilinguals, there were no significant correlations between ERPs in the time window around 140 ms on the left and any of the language measures or measures of language experience for the bilingual group as a whole. However, separated into the two subgroups (Spanish-dominant children, versus English-dominant bilinguals), the English-dominant children showed a strong positive correlation between the maternal language input and the right (T8) Ta-amplitude for the 140 ms time window (*r* = 0.81; p = 0.015). Specifically, more English input was correlated with more positive ERP responses.

The opposite effect was seen for the 10 Spanish-dominant children: In this group maternal input showed a negative correlation with right (T8) amplitude in the Ta-time window (140 ms) (*r* = -0.623; *p* = 0.054). In contrast, a negative relationship was observed between the Spanish language test scores and right amplitude (*r* = -0.627; *p* = 0.052). Also, input from the child’s siblings (8 children had siblings) showed a positive correlation at T8 (*r* = 0.98; p < .000), indicating that the more English heard from siblings, the more positive the ERP response.

On the left side at T7, there was a strong negative correlation in the Ta- time window for the first language exposure (10 children, *r* = -0.713; *p* = 0.021) and language input from the nanny or babysitter (9 children, *r* = -0.752; *p* = 0.019). Specifically, the more Spanish the child heard, the more positive the response at the left site.

For the Turkish-German children, there was a negative correlation between the father’s Turkish skills and the child’s amplitude at 140 ms (r = -0.75, *p* = 0.02) and at 160 ms (r = -0.75; *p* = 0.02) at the left site. Specifically, the better the father’s self-perceived Turkish skills, the more negative the amplitude in the Ta latency range. The receptive language measure in German showed a positive correlation with amplitude in the Tb-range at 200 ms (*r* = 0.65; *p* = .032); likewise, the pseudo-word repetition at 200 ms was positively correlated (r = 0.61, p = 0.044). Thus, children with better German showed more positive responses, which could indicate emerging Ta.

## Discussion

This study explored the left and right temporal ERP measures Na, Ta, and Tb to the English and German vowel phoneme /Ɛ / in monolingual and bilingual children. This vowel does not have phonemic status in the other language (Turkish or Spanish) of the bilingual children. Generally, all three peaks were easier to identify in monolingual versus bilingual children. The waveform morphology was generally comparable for the US and the German studies for the monolingual English and German children; however, the Ta and Tb peaks were identified in a higher proportion of children in the German than in the American study. This difference might be partially related to age and to design differences (e.g., headphones versus free-field presentation with load speakers). The Na-peak was clearly present for most children in all four groups. Ta was the most prominent peak in the German monolingual group at the right site while in the English monolingual group it was more well-defined at the left site. Tb was distinct in the German monolingual group at both left and right sites, but in the English monolinguals, only at the left site. The best model, using presence versus absence of temporal site peaks, revealed that bilinguals were more likely to have absent (i.e., poorly defined) peaks. In addition the best model of temporal-site amplitude suggested that a more negative amplitude was related to bilingualism of a child. This pattern is consistent with a general delay in maturation of the temporal site responses in both bilingual groups. Specifically, with increasing age, the Ta peak emerges, resulting in more positive amplitude. We did not see differences between groups in the morphology of the T-complex waveform (as determined by correlation with the monolingual grand mean). In addition, we found a number of correlations between the amplitude in the Ta-Tb latency range with language input. Interestingly, these correlations suggested a more complex relationship between language input and these temporal measures than anticipated. Below, we discuss these findings in relation to the prior literature.

### Effects of language experience on the temporal measures

We had predicted that increased experience with the second language would lead to T-complex responses more similar to the monolingual controls. However, the results revealed only partial support for this prediction. With more German input, better German receptive language skills and non-word repetition scores, Turkish-German children had responses more similar to German monolingual controls—seen specifically as a more positive amplitude. In addition, higher self-ratings of Turkish skills by fathers were negativity correlated with Ta amplitude. It is possible that a father with better Turkish skills is more likely to speak to his child in Turkish than German. However, Spanish-English bilingual children showed a more nuanced pattern related to the amount of Spanish input. For Spanish-English children who were clearly dominant in English, amount of English input correlated positively with Ta amplitude, similar to the pattern found for the German children. In contrast, for the children who received sufficient Spanish (balanced Spanish and English to more Spanish than English) and who were clearly functional in Spanish, amount of Spanish showed a positive relationship with the Ta amplitude measure. This pattern was also replicated when the first language or the language of the babysitter or nanny was considered. Interestingly, when examining the language a sibling spoke to the child, more English was associated with more positive responses.

One possible reason for this different pattern for the American compared to the German bilingual groups is that that the Spanish-English bilinguals in the American study ranged from balanced bilinguals to English dominant. Thus, most of the children were receiving substantial English input. The correlation may have reversed for the eight children with much more Spanish compared to the other bilingual children because acquiring strong skills in Spanish enhanced metalinguistic skills, including phonological awareness. These skills may have strengthened learning of English phonology [46]. It will be particularly interesting to examine whether these T-complex measures show a relationship to the development of skills such as phonological awareness. With regards to the bilingual children who were clearly dominant in English, it is possible that they are showing the opposite pattern because those who are receiving a small amount of Spanish, but not enough to enhance these general metalinguistic skills do not benefit from the bilingual experience. It is important to remember that these eight children all performed poorly on the Spanish vocabulary measure. In this case, receiving more English leads to better processing of English speech sounds.

An alternative outcome was that we would see increased negativity of the T-complex for monolingual, native listeners and for listeners who have more experience with the target language, as found in the only adult study that examined the affect of cross-linguistic experience on T-complex measures [33]. We did not see this pattern in the current study with children. The T-complex in adults was a well-defined sequence of peaks (Na-Ta-Tb) for both native (Polish) and non-native (English) listeners. It is possible that adults and children show different patterns because the Ta peak is only just emerging in children. It will be necessary to examine the T-complex in adult bilinguals to native versus non-native vowels to see whether adults and children show similar or different patterns.

### T-complex measures and language deficits

The current study examined typically-developing children who have been subjected to different types of language experience in their lives. The fact that bilingual groups show a comparatively reduced temporal site amplitude supports the idea that the T-complex may reflect poor (or reduced) phonological skills rather than being the cause of language deficits [14, 20]. More specifically, there was no evidence that the bilingual children in the current study have SLI. Thus, the finding of differences in the temporal site responses from monolinguals indicates that the learning experience is reflected by this measure. Thus, it is possible that children with SLI show poor temporal site responses because they are delayed in acquiring robust phonological categories compared to same-age controls. Indeed, both groups of children show smaller mismatch negativity (MMN) to vowel contrasts (/ε / versus /e/ in German [17] and /Ɛ/ versus /I/ in English [47]). The smaller MMN amplitude suggests poorer discrimination of the vowel contrast, at least for some of the bilingual children. In addition, the bilingual children did not differ in T-complex morphology from the monolingual controls, whereas many of the children with SLI showed poor waveform morphology (as determined by correlating each child’s waveform to the typical monolingual grand mean) [14]. Lower correlation values for some of the children in the current study were related to later peaks rather than an absence of peaks, whereas in the study of children with SLI a smaller amplitude Ta peak was related to poorer waveform morphology.

In a previous study with older children (6 to 11 years of age) a small proportion of typically-developing, monolingual children showed absent peaks or poor waveform correlations at temporal sites [14]. Thus, attenuated responses at temporal sites do not necessarily correspond to poor language. We still know very little about the normal range of phonological development in relation to neural measures. We also do not know how these measures relate to pre-literacy skills, such as phonological awareness. The children in the current study were in the year before school, and thus, many of them were only just beginning to develop skills related to reading (such as learning the alphabet). We will return to this issue when we discuss the relationship of the neural measures to language input.

### Developmental aspects of temporal site responses

In the current study, the German monolinguals show the most clearly-defined peaks at the temporal sites. The children in the German study were older than those in the American study by approximately one year. Previous studies indicate that the individual T-complex peaks mature at different rates [22, 31]. The Na amplitude and latency have been shown to decrease from five to 20 years of age [31] although the rate of decrease is much more rapid from one to four years of age, and slows considerably from four to seven years of age [22]. Na can be reliably elicited in children from as early as 18 months of age and there is some possibility that Na partially reflects the opposite pole of the frontocentral P1 peak [22]. Further studies will be necessary to establish the Na sources, but regardless of the outcome, these studies indicate that Na can be used to examine maturation of auditory processing in very young children.

The bilingual children in the current study were of comparable age to their respective monolingual controls. They also showed relatively high percentages of identifiable Na peaks (80–100%), except at the left site for the Spanish-English group (56%). Na peaks were also less clear at the left than the right sites for all groups and this pattern has been observed in other studies [14, 20, 22, 31]. It is possible that differences in orientation of the sources underlying the left and right sites lead to this difference. Future studies will be necessary to determine whether this difference is structural in nature (i.e., orientation of the sources) or functional.

With regards to the Ta peak, the monolingual German children that were also the oldest children showed the largest Ta amplitude of all groups and a more prominent peak than observed in the younger monolingual US children. This peak was more prominent on the right. This increase in right-hemisphere positivity has been observed in previous studies [14,20,22]. The Ta peak amplitude did not correlate with age in the current study, but the age range may have been too narrow to observe this relationship.

In addition, the study by Shafer et al. [22] showed no consistent increase in the presence of Ta from four to seven years of age (and the four and five year old monolinguals are the same children from this study). In fact, the Ta was found for many children at four years of age, but less-so for children at five and six years of age, but then, again clearly observed at seven years of age [22]. It is possible that these changes are related to the maturation of other sources that lead to masking of the Ta. However, it was clear that the Ta amplitude generally increased in positivity from three years of age compared to older ages (four to seven years) in Shafer et al. [22]. These findings suggest that maturation of the underlying sources leads to increased positivity, even though a clear Ta peak cannot always be identified. Identification of the Ta peak is also dependent on the following negative-going deflection (Tb). So it is possible that small or absent Tb leads to the absence of a distinct Ta peak. Tonnquist-Uhlén et al. [31] did not find an age-related decrease in Ta-peak latency at electrodes T7 and T8 in children ranging from five years of age up to adulthood. They also did not find a consistent age-related pattern for Ta-amplitude. Another study observed Ta-amplitude to decrease with age [24] but this study examined much older children.

With regards to the Tb peak, for the American study, the Tb peak was not reliably present in the target time-range. Only 65% of the monolingual English children showed an identifiable peak, and less than half of the bilingual Spanish-English children showed a peak. In contrast, in the monolingual German children all but two of the children showed identifiable peaks. However, only slightly more than half of the Turkish-German children showed a Tb peak at the left site.

One study suggests that the Tb is reliably present in older children (10 years and more) [24]. However, this study used a mastoid reference and the prominent Tb may actually be the inversion of the fronto-central P2 peak. Tonnquist-Uhlén et al. [31] reported no age-related changes in Tb-latency but they did see a decrease in Tb-amplitude on the right side with increasing age. In this case, they were focusing on the age range from five years to adulthood, and collapsed across five and six year olds. One factor that might account for the difference in the presence of a clear Ta and/or Tb peak between the US and German study may be stimulus delivery. Stimulus delivery over headphones or ear inserts is likely to minimize ambient background noise that can be found even in sound-attenuating booths. Bruneau et al. [29] found that less intense sounds led to smaller and later latencies of the Tb (which they called the N1c).

Manipulation of stimulus and task factors, such as intensity [29] or interstimulus interval [48] will help explain these maturational patterns. In particular, if Ta and Tb reflect different functions/sources, then manipulation of stimulus/task factors may allow modulation of the functions in such a way to help explain the absence of Ta-Tb peaks in some children.

### Functional significance of the T-complex peaks

Reduced T-complex measures may thus have multiple causes: In the case of language-impaired children, the reasoning is that these children might have poor speech or auditory processing skills that is general in nature [14]. In the case of the children with bilingualism, however, there is no reason to claim that they have generally poor auditory/speech processing, since they have acquired phonology in the first language. In addition, the target [Ɛ] stimulus can be treated as a phoneme in any of the languages. One possible reason for differences in bilinguals is that these speech vowels are better exemplars for German and English. Thus, even though these speech sounds can be assimilated into a Turkish or Spanish phoneme category, they are not perceived as good exemplars. In this case, the Ta-Tb peaks may reflect a sharper neural representation. Another possible explanation for the differences in bilingual and monolingual groups is that bilingual listeners may allocate resources to processing speech differently than monolinguals because they need to also determine which language the speech belongs to. A third possibility is that some of the bilingual children are not receiving robust language input for either language. Future studies will be necessary to decide between these accounts. One approach will be to manipulate the language-specific “goodness” of the speech stimulus. Another approach will be to more closely examine the parents/caretakers’ language skills in relation to the child’s speech perception.

### Limitations

These two studies were designed independently of each other, and thus, did not use identical tools for addressing the questions. Even so, the pattern of findings was remarkably similar despite these differences. The two child groups differed with respect to social, linguistic and educational factors, as well as age. In particular, the Spanish-English group was more heterogeneous that the Turkish-German group, possibly in part due to the diverse setting of New York City compared to a small town in Germany.

We chose to compare children who were in preschool or just entering school because we expected similarities in educational experience. In the first year of school (Kindergarten in the US and first grade in Germany) children in both countries receive considerable instruction in pre-literacy skills in the dominant language (English or German). Preschool is not mandated in either country; thus, there is variability in whether children received this experience. Despite the possibility that some of the differences across studies were related to maturation, the patterns across the bilingual and monolingual groups for the two studies were remarkably similar.

## Conclusion

This study found that the AEP responses measured at the temporal sites were modulated by language experience. Na, Ta and Tb peaks were less-well formed and more negative in amplitude in many of the bilingual children in the current study. It is unclear why this is the case, but the pattern of relationships between amplitude of the AEP at these temporal sites and language input factors suggests a complex relationship. More experience with the target language is generally beneficial. However, we also found that having balanced input in both languages for the Spanish-English bilinguals led to more “mature” responses that resembled the monolingual controls. Future studies using larger groups are necessary to clarify which factors are responsible for the development of robust temporal site AEP responses to speech. In addition, it will be important to clarify why many children with SLI show poorer AEP responses at these temporal sites. It will also be interesting to determine whether targeted phonological training can improve neural responses to language-specific speech at the temporal sites in children who are learning a second language or in children with SLI.
